# Molecular mechanism of aerobic exercise in miRNAs and the anti-tumoral effects

**DOI:** 10.3389/fonc.2025.1595982

**Published:** 2025-08-13

**Authors:** Merve Çulha, Fabio S. Lira, Jose C. Rosa-Neto

**Affiliations:** ^1^ Immunometabolism Lab, Institute of Biomedical Sciences and Faculty of Medicine from University of São Paulo, Graduate Program in Oncology, São Paulo, Brazil; ^2^ Exercise and Immunometabolism Research Group, Post-graduation Program in Movement Sciences, Department of Physical Education, Universidade Estadual Paulista (UNESP), São Paulo, Brazil

**Keywords:** exercise- induced miRNAs, cancer, aerobic exercise, anti- tumoral effects, immunotherapy

## Abstract

Exercise decreases cancer risk, prevents recurrence and enhances treatment efficacy, however its exact mechanisms remain unclear. This review aims to (i) summarize the miRNAs released during aerobic exercise and explore the factors influencing their levels; (ii) discuss the miRNAs associated with cancer and exercise. Exercise alters circulated-miRNA levels. Some of them show benefits on tumor and anti-tumoral immune cells (miRNA-1, miRNA-133, miRNA-206, miRNA-208, miRNA-486, miRNA-499) and others exert adverse effects (miRNA-21, miRNA-221). Mechanisms of exercise effects on cancer are complex due to some exercise- induced miRNAs exert advantageous, others not, individual characteristics and exercise features influencing the miRNAs levels. Further studies are needed to elaborate the exercise features on immune response and tumor cell functions, including miRNA production.

## 1 Introduction

Cancer is the second leading cause of death worldwide. The estimates suggest that, within the next decade, cancer may become the leading cause of death. Several factors contribute to this rising trend, including increased life expectancy (aging), the consumption of industrialized and high-calorie foods, and a sedentary lifestyle. These elements collectively contribute to the increasing incidence of cancer (1). The shift towards a more sedentary lifestyle has progressively reduced individuals’ physical activity levels, leading to decreased functional capacities and increased physical inactivity (2). It is well known that exercise influences metabolic regulation by altering the secretion of cytokines, myokines, and microRNAs (miRNAs), thereby strengthening the immune system and reducing the risk of various types of cancers, such as breast, lung, prostate, and colon cancers (3, 4). Though the existing literature provides information on the effects of exercise on cancer progression and the role of miRNAs in cancer, there is a lack of studies specifically addressing the impact of exercise-induced miRNAs on both cancer progression and the immune system. This review aimed to explore the effects of aerobic exercise-induced miRNAs on tumor cells and antitumor immune cells.

### 1.1 The biology of miRNAs

**Figure 1:**
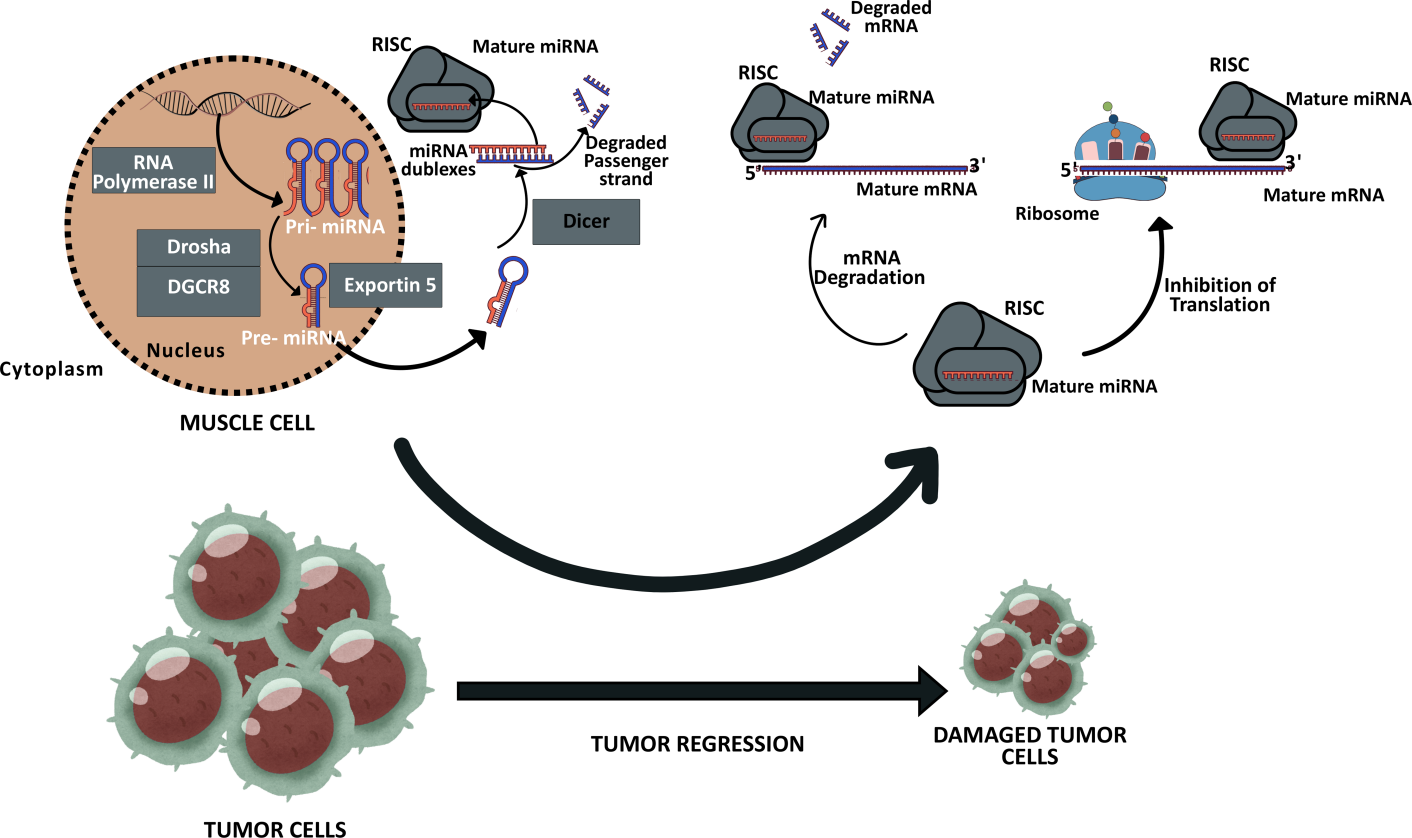
Expression of MyomiRs in the muscle cell and these MiRNAs possible effects on the tumor cells. Muscle cells are capable of secreting specific miRNAs, which can undergo modification in response to exercise and are designated as MyomiRs. Upon reaching target cells, including tumor cells, these miRNAs have the potential to influence their fate, thereby contributing to the modulation of cancer progression. Consequently, the regulation of these MiRNAs’ expression levels might hold significant therapeutic potential in the fight against cancer. The new figure was created using Canva.

The biogenesis of microRNAs (miRNAs) can occur via two distinct pathways: canonical and noncanonical (5). In the canonical pathway, a precursor miRNA (pri-miRNA) with an elongated hairpin structure is transcribed in the nucleus from the corresponding DNA region by RNA polymerase II. This pri-miRNA is then processed into a pre-miRNA by cleavage at the base of the hairpin by a microprocessor complex, which includes Drosha (an RNase III enzyme) and DiGeorge critical region 8 (DGCR8), which assists in Drosha’s function. The resulting pre-miRNAs are transported from the nucleus to the cytoplasm by Exportin 5. In the cytoplasm, the terminal loop of the pre-miRNA is then removed by another RNase III enzyme, Dicer, which produces miRNA duplexes that are 21–24 nucleotides in length (6). In the final stage, the mature miRNAs are loaded into the RNA-induced silencing complex (RISC) containing Argonaute (AGO) as a core component, Dicer, and the double-stranded RNA-binding protein TRBP; the newly formed miRNAs may now display their functions on mRNAs (7) (**Figure 1**).

In noncanonical pathways, the same proteins can assume different roles with varying compositions. Two notable examples of these noncanonical pathways are the “Drosha-DGCR8-independent pathway” and the “Dicer-independent pathway.” In the Drosha-DGCR8-independent pathway, pre-miRNAs such as mirtrons are generated. These structures are transported to the cytoplasm by Exportin 1 without the involvement of Drosha, as they are already similar to Dicer products (5). In the Dicer-independent pathway, Drosha cleaves short hairpin RNAs and the obtained pre-miRNAs are directly loaded into AGO2 because they are not of the proper length for Dicer processing. Generally, while the 3p strand activity is suppressed, the 5p strand matures due to AGO2 cleavage activity (5, 8).

These expressed miRNAs can exhibit autocrine, paracrine, and endocrine effects when released into the extracellular space or body fluids (9). They can be transported via exosomes, microvesicles, apoptotic bodies, high-density lipoproteins, and ribonucleoproteins (9, 10). Due to their paracrine and endocrine properties, miRNAs play a critical role in diseases, including various types of cancer, and in regulating numerous biological processes, such as apoptosis, cell proliferation and differentiation, stem cell growth, and immune system regulation (11). Notably, the expression of miRNAs can be influenced by lifestyle factors such as diet (12) and exercise (13, 14). When miRNAs are induced by exercise and secreted from muscle tissues, they are referred to as “myomiRs.” Some myomiRs are associated with cancer inhibition by suppressing oncogene expression or tumor cell proliferation (15, 16) (**Figure 1**).

### 1.2 Effects of acute aerobic exercise on miRNA expression and secretion

Epidemiological reports have demonstrated that exercise reduces the risk of various illnesses, including diabetes (17), neurodegenerative disorders (18), metabolic diseases (19), age-related conditions (20), and cancer (21). Additionally, exercise positively impacts mitochondrial metabolism, cardiovascular health, and the immune system (22, 23). These benefits may be attributed to the secretion of extracellular vesicles (EV), containing myokines, growth factors, chemokines, and miRNAs, from muscle and other tissues and their contribution to the communication of distanced organs (24–28).

Exosomes, microvesicles, and apoptotic bodies are regarded as EV. Microvesicles, which are approximately 100 nm to 1 µm in diameter, bud off from cells and have a droplet-like appearance (11, 29). In contrast, exosomes are smaller, typically ranging from 30 to 100 nm (30) or 80 to 120 nm (31), and are formed by the fusion of endosomal membranes with the plasma membrane.

Notably, miRNAs may freely circulate extracellularly due to their affinity for lipoproteins, which enhances their stability and resistance to pH changes, RNase activity, and temperature fluctuations (5, 32, 33).

Besides the evidence suggesting that acute aerobic exercise is able to induce changes in miRNA expression in skeletal muscle tissue and blood (34, 35), a preclinical study showed that regular aerobic exercise can modify miRNAs in extracellular vesicles (35). They observed a 35% reduction in prostate tumor size, accompanied by modifications in miRNA concentrations and without significant differences in EV size after exercise. This report indicates that even a slight increase in miRNA levels in the EV can have significant effects on tumor cells.

## 2 Discussion and conclusion

According to previous studies, fluctuations in miRNA levels in the blood are associated with tumor malignancy. This evidence strongly indicates that exercise-induced miRNAs may be promising for cancer therapy as they inhibit tumor growth. As found in the literature, miRNA-1, miRNA-133, and miRNA-206 have come into prominence in this focus (36–38).

### 2.1 MiRNA-1

MiRNA-1 is a cancer suppressor gene, and its expression level decreases in different cancer types, such as head and neck squamous carcinoma as well as in thyroid, lung, cervical, and colorectal cancers (36, 37). The importance of miRNA-1 may depend on its relationship with many cell functions and pathways, such as DNA replication, cell division, DNA repair, G1/S transition of the mitotic cell cycle, and the p53 signaling pathway (39). Tumor cells show reduced viability when a combination of miRNA-1 and anticancer drugs are used for treating lung or colorectal cancer (40). These reports demonstrate that increasing miRNA-1 expression levels may be beneficial against cancer. However, some studies show that miRNA-1 inhibitors promote cancer cell apoptosis, decrease both the proliferation and migration of hepatocellular carcinoma cells, and reduce lung cancer sensitivity to anticancer drugs. Moreover, the use of miRNA-1 inhibitors has some disadvantages given that they modify the tumor microenvironment, inhibiting the migration ability of monocytes and T cell infiltration (37, 41). Therefore, examining the effects of miRNA-1 on each cancer type and immune cells may be fundamental for using miRNA-1 in an efficient way.

### 2.2 MiRNA-133

MiRNA-133 is effective in the treatment of some cancer types, acting over the PI3K/AKT (38) or CDC42/PAKs pathways (37). In addition, miRNA-133 displays lower expression levels in cancer stem cells (42). Importantly, increased miRNA-133 levels restrict the migration and proliferation of some cancer cells, such as bladder and prostate cancer, lung cancer, and gastric cancer cells, by exerting action over a variety of targets (for example, COL1A1 mRNA, EGFR, and FOXC1) (43–45). Clinical reports have demonstrated an increase in serum miRNA-133 levels along with an improvement in white blood cell counts following chemotherapy (37). Additionally, some studies suggest that miRNA-133 may enhance the functions of NK cells and CD8^+^ T cells by modulating HLA-G expression in cancer cells, thereby promoting a more effective immune response in TME (46, 47). These findings imply that elevated miRNA-133 levels are crucial for the survival of patients with cancer.

### 2.3 MiRNA-206

MiRNA-206 is a Wnt oncogene suppressor (38) with several beneficial effects to restrict a variety of tumors, like osteosarcoma, endometrioid adenocarcinoma, breast cancer, colorectal cancer, lung cancer, and hepatocellular carcinoma (48–56). MiRNA-206 expression can inhibit both differentiation and migration of colorectal cancer cells by targeting TM4SF1 (51), reduces invasion through lnc00152 (52), and increases patient survival (54). In addition, colony formation in non-small cell lung cancer or tumor cell migration and proliferation can be diminished by miRNA-206 due to a reduction in c-Met and Bcl2 levels (53). Moreover, combined treatments including miRNA-206 and curcumin impair the PI3K/AKT/mTOR signaling pathway, which is involved in lung cancer development (55). miRNA-206 may exert both advantageous and disadvantageous effects depending on the cancer type. For example, it has been reported to reduce apoptosis in metastatic breast cancer cells, whereas in hepatocellular carcinoma, it enhances apoptosis and suppresses c-MET expression during tumor initiation (56). Furthermore, in hepatocellular carcinoma, miRNA-206 contributes to an improved immune response by promoting M1 macrophage polarization and facilitating CD8^+^ T cell infiltration into the tumor microenvironment (TME) via modulation of CCL2/CCR2 signaling. These findings suggest that miRNA-206 may serve as a promising therapeutic target in cancer treatment (57).

### 2.4 MiRNA-208

Most studies indicate that miRNA-208 contributes to cancer progression. Liu et al. reported that miRNA-208 promotes metastasis by inducing EMT in pancreatic cancer (58). Similarly, miRNA-208 has been associated with increased proliferation, invasion, tumorigenesis, and metastasis in melanoma, hepatocellular carcinoma, and esophageal squamous cell carcinoma (59–61). Although some evidence suggests that miRNA-208 may suppress invasion in lung cancer via the STAT3 pathway (62), its overall role appears to favor tumor development. Additionally, miRNA-208 impairs anti-tumor immunity by promoting Treg cell expansion (63). Thus, targeting miRNA-208 might offer therapeutic benefit in cancer.

### 2.5 MiRNA-486

The effects of miRNA-486 on cancer appear to be context-dependent. In several cancers, it shows tumor-suppressive roles by inhibiting cell proliferation, migration, invasion, glycolysis, and stemness. For instance, miRNA-486 suppresses tumor progression in gastric (64) colorectal (65, 66) cervical (67) and lung cancers (68) by targeting genes such as FGF9, NEK2, PIK3R1, ECM1, and GAB2. In contrast, it can act as an oncogene in ovarian and pancreatic cancers by promoting proliferation and invasion through regulation of CADM1 and PTEN (69, 70). Furthermore, miRNA-486 may influence immune responses by modulating MDSCs and TILs in lung cancer, thereby contributing to either tumor progression or suppression (71, 72).

### 2.6 MiRNA- 499

miRNA-499 may exert dual roles in cancer progression, similar to miRNA-208 and miRNA-486. It has been shown to suppress tumor proliferation and metastasis by targeting VAV3 in endometrial and lung cancers, and to inhibit tumor growth in colon cancer (73–75). Conversely, some studies reported that miRNA-499 may promote colon cancer progression by downregulating tumor suppressor genes such as PDCD4 and FOXO4 (76). Although no direct evidence links miRNA-499 to T or NK cell regulation, Wang et al. showed it may promote M1 to M2 macrophage transition, potentially impairing anti-tumoral immunity (77).

Aerobic exercise increases miRNA production and secretion. Especially, acute aerobic exercise can elevate the expression of miRNA-1, miRNA-133, and miRNA-206. Nielsen et al. (35) showed that all miRNA levels are reduced in plasma immediately after acute endurance exercise, but miRNA-1, miRNA-133a, and miRNA-133b elevate 3 h later. Similarly, Russell and Lamon (78) found increased miRNA-133a levels in muscle tissue after 3 h. Moreover, during downhill walking, they observed an increase in miRNA-1, miRNA-133a, and miRNA-133b levels between 2–6 hours post-exercise (79). These studies show that the expression of muscle-specific miRNAs (myomiRs) is easily modified by exercise, but it may take time to release these myomiRs into the bloodstream after they are expressed.

Based on these specific miRNAs, miRNA-208, miRNA-486 and miRNA-499 which are upregulated and downregulated by exercise. Although current findings on the response of miRNA-208 to aerobic exercise remain inconsistent, several studies report an increase in its levels following exhaustive aerobic activities, such as marathon running (80, 81). This transient elevation is likely attributable to cardiac muscle cells, which are the primary source of miRNA-208. Conversely, Soci et al. reported a downregulation of miRNA-208 expression in response to chronic aerobic training (82). These results suggest that low- to moderate-intensity acute exercise, or long-term aerobic exercise protocols, may help avoid potentially undesirable increases in miRNA-208 expression, especially considering its pro-tumoral effects reported in certain cancer types. Similarly, miRNA-499 is predominantly expressed in cardiac and skeletal muscle tissue. While its circulating levels are not significantly altered by moderate- or high-intensity aerobic exercise in healthy individuals (83–85), prolonged endurance activities can induce a transient increase in its expression in both trained and untrained individuals (33, 86).

In addition to the evidence regarding the association between exercise, exercise-induced miRNAs, and tumor cells, aerobic exercise has been reported to influence antitumor immune cells incertain aspects. For instance, aerobic exercise directly affects the number, functions, and mobilization of immune cells (87). Oliveira et al. (88) and Pudkasam et al. (89) found that 1 h acute aerobic exercise at 70% VO2 enhances the function of neutrophils and results in 5 times more NK cells, which are key for cell defense against pathogens and play a crucial role in cancer immune surveillance. Similarly, Bigley et al. (90) determined that NK cell and NK subset levels elevated immediately after exercise. Additionally, Fairey et al. (91) found that, among cancer survivors, the cytotoxic activity of NK cells increased following exercise. Beyond enhancing the activity and number of NK cells, aerobic exercise has also been found to significantly increase the number of CD4+ and CD8+ T cells after 1 h, further demonstrating its anti-tumoral benefits (92). Given that imbalances in the CD4+/CD8+ T cell ratio lead to decreased immune defense (93), the observation that aerobic exercise increases the number of the aforementioned immune cells and their ratio (94) suggests the potential of aerobic exercise as a therapeutic approach for cancer risk and progression. In addition to these benefits, the anti-tumoral defense mechanism of exercise is linked to its ability to reduce inflammation and improve the blood lipid profile, resulting in mature NK cells and the activation of T lymphocytes. Based on existing literature, aerobic exercise has been shown to influence circulating miRNA levels in the blood. When upregulated, certain miRNAs—including those mentioned earlier—may exert antitumor effects (38, 95). However, a comprehensive review of the scientific literature revealed no direct studies investigating the impact of aerobic exercise–induced changes in miRNA expression on antitumor immune cells. Interestingly, Nguyen et al. (96) identified several miRNAs, such as miRNA-21—whose expression is modulated by exercise (96)—that can alter antitumor immune cell functions. MiRNAs like miRNA-155 support immune cell activation (97), while miRNA-221 enhances T cell survival by reducing apoptosis through downregulation of CDKN1B expression (98). Moreover, miRNA-155 regulates the differentiation of CD4+ T cells into Th1, Th2, and Th17 subsets, and influences CD8+ T cell effector functions (99, 100). However, the levels of these miRNAs in circulation may vary depending on the exercise protocols; for example, a report showed that miRNA-221 expression is reduced in NK cells during aerobic exercise (96). Therefore, understanding miRNA expression variations during exercise sessions is crucial in the development of new immunotherapies and cancer therapies.

Additionally, a few studies related to aerobic exercise and exercise-induced miRNAs expression in lymphocytes were found. One of these studies showed that acute aerobic exercise resulted in changes in the expression of 23 miRNAs in NK cells, and 5 of these miRNAs were associated with melanoma, glioma, focal adhesions, and prostate cancer (101). Notably, following a half-marathon, patients diagnosed with breast, colon, and prostate cancer displayed an upregulation in NKG2D expression, which may change tumor progression through exercise-driven epigenetic modifications (21). These reports imply that exercise can modify miRNAs in antitumor immune cells and affect cancer progression through the action of such miRNAs. In support of this claim, Chilton et al. (92) revealed changes in the expression of 56 miRNAs in leukocytes after acute aerobic exercise; 4 of these miRNAs were associated with telomeric gene mRNAs, indicating that aerobic exercise may lead to longer leukocyte telomeres through miRNA-mediated epigenetic regulation, enhancing the immune defense against cancer. In contrast, after an ultra-long-distance marathon, mRNA expression showed no variation (98), suggesting that miRNA levels were not altered either. Therefore, analyses on exercise-induced miRNAs in the circulation following each exercise protocol and their effects on tumor cells and antitumor immune cell functions in a certain cancer type are needed to advance in the development of both immunotherapy and cancer therapy. Future studies might encompass additional objectives. For instance, further elucidation of exercise-induced miRNA effects on specific immune cell subclasses that exhibit intricate interactions with one another is required. In addition, exercise-induced miRNAs should be combined for testing on tumor or antitumor immune cells *in vitro*, because these cells are exposed to multiple exercise-induced miRNAs in the human body. Finally, exercise protocols incorporating both aerobic and resistance training should be tested to expand the range of miRNAs expressed and to evaluate their effects on the tumor microenvironment.

In summary, exercise exerts beneficial effects on cancer prevention. MiRNAs play crucial roles in this amelioration process; however, the mechanisms underlying the benefits of exercise-induced miRNAs in cancer treatment remain unclear. Some of the reasons for this may be the influence of various factors on the expression of exercise-induced miRNAs, including the type, duration, and intensity of exercise, as well as individual characteristics such as sex, age, and exercise background. While some miRNAs have been observed to have beneficial effects on tumor cells, others exert adverse effects on antitumor immune cells. This discrepancy can be a significant challenge in the treatment of cancer using exercise. Furthermore, each cancer cell type exhibits a different response to a specific miRNA. Consequently, understanding how exercise upregulates or downregulates miRNA levels and the subsequent influence of exercise-induced miRNAs on tumors and antitumor immune cells is complex. Elucidation of the exercise axis, exercise-induced miRNA levels, and their effects on tumor cells and antitumor immune cell function according to cancer type is necessary to support cancer therapy and immunotherapy.
